# Removal of Plaster-Of-Paris Bandages

**Published:** 1911-04-29

**Authors:** 


### EDITOR'S LETTER-BOX.

### REMOVAL OF PLASTER-OF-PARIS BANDAGES.

A Medical Correspondent writes as follows : A short article on the above subject appeared in your issue of March 25, and it appears to be the report of the Transactions of an Austrian Society of Medicine. The members of that society had the advantage of hearing one of their number expound some views on elementary chemistry which strike one as being particularly misleading, but at the same time harmless and trivial in character. The report states that the method is based on a fact familiar to students of elementary chemistry that calcium carbonate is decomposed by acids with liberation of carbonic acid gas, to which statement we will agree. We may also add for the benefit of students of elementary chemistry that plaster of Paris and calcium sulphate are synonymous terms and that this chemical body is entirely insoluble, unaffected, and much less destroyed by acetic acid. This fact would probably elucidate the problem of why this process, though so simple, was not thought of before. Possibly either by accident or design some calcium carbonate, otherwise known by the much simpler term of "chalk," had got mixed up with the plaster belonging to the "Casual Communicant," which accounts for the evolution of gas, and incidentally softened the line of separation on the splint. He would have done equally well if he had used hot water and let it soak well into the plaster, for although this method has no pretensions to originality or to any subtle chemical reaction, elementary or otherwise, it is effective in that the plaster being well soaked with water cuts easier than when it is dry. The acetic acid may have some action on the material of which the bandage is made, as it is well known that mineral acids (nitric or sulphuric) are highly destructive to cotton fabrics, but even then you have to get through the plaster to get to the layers of bandage.

